# A R3-Type MYB Transcription Factor LrMYB30 Negatively Regulates L. *ruthenicum* Fruit Coloration

**DOI:** 10.3390/genes16121501

**Published:** 2025-12-15

**Authors:** Yuejie Wang, Tingting Wang, Zhanming Tan, Zixin Mu

**Affiliations:** 1 State Key Laboratory Incubation Base for Conservation and Utilization of Bio-Resource in Tarim Basin, Tarim University, Alar 843300, China; wangyuejie2019@163.com; 2 College of Life Science & Technology, Tarim University, Alar 843300, China; 3 College of Grassland Agriculture, Northwest A&F University, Yangling 712100, China; 4 College of Food Science and Engineering, Tarim University, Alar 843300, China; tingtingwangedu@163.com; 5 College of Horticulture and Forestry, Tarim University, Alar 843300, China; tlmdxtzm@taru.edu.cn

**Keywords:** MYB transcription factor, fruit coloration, *Lycium ruthenicum* Murr., anthocyanin metabolism, proanthocyanidins

## Abstract

**Background:** Anthocyanins and proanthocyanidins (PAs), as flavonoid compounds with potent antioxidant activity, exhibit significant health-promoting and medicinal properties. Black wolfberry (*Lycium ruthenicum* Murr.) is renowned for its exceptional anthocyanin content; however, the regulatory mechanisms of anthocyanin biosynthesis remain poorly understood, limiting its biotechnological potential. This study aimed to elucidate the transcriptional regulatory function of *LrMYB30* in anthocyanin biosynthesis in black wolfberry. **Methods:** The regulatory function of *LrMYB30* was investigated using virus-induced gene silencing (VIGS), yeast one-hybrid assays, and dual-luciferase reporter assays in black wolfberry. **Results:** VIGS demonstrated that silencing *LrMYB30* promoted anthocyanin accumulation while reducing PA content, establishing that the LrMYB30 transcription factor as a negative regulator of anthocyanin synthesis. Yeast one-hybrid and dual-luciferase reporter assays confirmed that LrMYB30 directly binds to and activates the promoter of *LrANR*, a key structural gene in PA biosynthesis. In contrast, LrMYB30 neither binds to nor suppresses the promoters of the critical anthocyanin biosynthesis genes *LrUF3GT* and *LrDFR*. **Conclusions:** Thus, LrMYB30 redirects the flavonoid metabolic flux from anthocyanin to PA synthesis through transcriptional activation of *LrANR* during later fruit development, reducing anthocyanin accumulation and delaying coloration. These findings reveal a novel regulatory mechanism in black wolfberry pigmentation and maturation, providing genetic targets for molecular breeding of high-anthocyanin cultivars.

## 1. Introduction

The chromatic attributes of flowers and fruits represent adaptive traits that have evolved to attract pollinators and seed dispersers, thereby enhancing reproductive fitness. This pigmentation arises predominantly from phytochemicals including chlorophyll, glycosylated anthocyanins, and carotenoids [1]. As water-soluble vacuolar pigments, anthocyanins exhibit pH-dependent color shifts, generating spectral variations ranging from red to blue hues. Their biosynthesis typically coincides with maturation stages, establishing them as robust biomarkers for fruit ripening. Proanthocyanidins (PAs), or condensed tannins, significantly modulate organoleptic characteristics through effects on flavor profiles, textural properties, and astringency perception [2]. Notably, both anthocyanins and PAs demonstrate potent antioxidant capacities with documented beneficial impacts on human health, encompassing anti-inflammatory, anti-carcinogenic, immunomodulatory, and cardioprotective functions [3]. Given these nutritional attributes, these secondary metabolites have emerged as principal targets for plant metabolic engineering and synthetic biology applications [4,5].

Anthocyanin biosynthesis is underpinned by a complex regulatory network involving endogenous developmental programs and environmental cues. Endogenous regulation encompasses ontogenetic processes and hormonal signaling pathways, while environmental influences incorporate light intensity and quality, temperature fluctuations, salinity levels, UV radiation exposure, nitrogen availability, and drought stress [1,6]. Phytohormone-mediated signaling pathways serve as pivotal regulatory nodes in both climacteric and non-climacteric fruit development systems. Specifically, abscisic acid (ABA), ethylene, and jasmonic acid (JA) have been mechanistically validated as positive regulators of anthocyanin biosynthesis [7,8,9], whereas auxin, gibberellins (GA), and nitric oxide (NO) exert inhibitory control over pigment accumulation [10,11,12,13,14].

Current research identifies the MYB–bHLH–WD40 (MBW) transcriptional complex as the core regulatory module that orchestrates anthocyanin biosynthesis at the transcriptional level [5,15,16]. Within this tripartite complex, MYB proteins confer substrate specificity by forming heterodimers with bHLH transcription factors and subsequently binding to promoters of biosynthetic genes. Beyond their established role as transcriptional activators, certain R2R3-MYB and R3-MYB members also function as repressors through competitive inhibition mechanisms [17]. Despite comprehensive characterization of MBW complex components across multiple plant species, three critical knowledge gaps persist: (1) elucidation of signaling cascades transducing environmental stimuli to MBW activation; (2) molecular mechanisms governing phytohormone-mediated regulation of this complex; and (3) functional dynamics between MYB activators and repressors [18].

The flavonoid biosynthetic pathway serves as the biochemical nexus for anthocyanin and PA production, with these compounds representing divergent branch-specific metabolites. The core pathway enzymes—including the scaffold-forming enzymes chalcone synthase (CHS) and chalcone isomerase (CHI), the hydroxylation catalysts flavanone 3-hydroxylase (F3H), flavonoid 3′-hydroxylase (F3′H), and flavonoid 3′,5′-hydroxylase (F3′5′H), and the reductive enzyme dihydroflavonol 4-reductase (DFR)—catalyze sequential reactions culminating in the synthesis of leucoanthocyanidin, a pivotal metabolic intermediate, with this functional categorization reflecting the modular organization that enables precise regulation of flavonoid structural diversity and accumulation in planta. This precursor undergoes divergent conversion through two distinct enzymatic routes: leucoanthocyanidin reductase (LAR) facilitates its transformation into (+)-catechin, while anthocyanidin synthase (ANS) oxidizes it to anthocyanidin. The latter intermediate can be further processed via anthocyanidin reductase (ANR) to yield (–)-epicatechin or undergo glycosylation by UDP-glucose:flavonoid 3-O-glucosyltransferase (UF3GT) to enable the subsequent biosynthesis of stable anthocyanins [1,15,19]. Notably, these competing branches exhibit precursor allocation dynamics, with documented antagonism between PA and anthocyanin biosynthesis in species for instance, *Medicago truncatula*, *Arabidopsis thaliana*, *Pyrus* spp., and *Actinidia deliciosa* [20,21,22]. The enzymatic competition between ANR (PA biosynthesis) and UFGT (anthocyanin biosynthesis) for leucoanthocyanidin constitutes a critical regulatory node in flavonoid metabolism [23,24].

*Lycium ruthenicum* Murr., a xerophytic shrub with ecological importance in desertification control, exhibits exceptional anthocyanin concentrations in mature berries [25]. These pigments serve as the primary chromatic determinants of its fruit coloration [26], with developmental-stage-specific color transitions correlating precisely with spatial-temporal patterns of anthocyanin deposition governed by biosynthetic gene expression dynamics [27]. Despite extensive characterization of its pigment profile, the transcriptional regulatory mechanisms remain incompletely defined compared to other berry crops [28,29,30]. While functional MYB activators have been identified [31,32,33], no inhibitory regulatory factors have been mechanistically validated to date. Our transcriptomic analyses revealed that the R3-MYB transcription factor LrMYB30 displays inverse correlation with anthocyanin accumulation, yet positively correlates with both NO signaling and PA biosynthesis [13]. This investigation seeks to mechanistically characterize LrMYB30’s role as a transcriptional repressor in anthocyanin biosynthesis. Our findings are anticipated to uncover novel MYB regulatory paradigms and highlight evolutionary divergence in anthocyanin metabolic control across plant species.

## 2. Materials and Methods

### 2.1. Plant Materials

The field trial was implemented at the Wolfberry (*Lycium*) Germplasm Repository of Ningxia Academy of Agriculture and Forestry Sciences (38°38′ N, 106°09′ E; elevation 1100 m), located in the Ningxia Hui Autonomous Region, China. Mature fruits of Black goji were selectively harvested from five-year-old plants cultivated under standardized management conditions.

### 2.2. Anthocyanins Measurements

The total anthocyanin content in black wolfberry fruit extracts was measured spectrophotometrically as described by Li et al. [7]. Quantification was based on a calibration curve established using cyanidin-3-glucoside (Cat. No. 626B021, Solarbio, Beijing, China) as the reference standard. The analysis was conducted with three biological replicates, each consisting of a pool of at least 30 fruits.

### 2.3. Proanthocyanidins Determination

The proanthocyanidin (PA) content in black wolfberry fruits was quantified using high-performance liquid chromatography (HPLC) as described by Li et al. [13]. Quantification was achieved by referencing a standard curve constructed from the peak areas of known concentrations of (+)-catechin. Proanthocyanidin content was quantified via HPLC and expressed on a fresh weight basis as mg/g FW. To mitigate biological variability, each of the three biological replicates was constructed from a composite sample of at least 30 fruits.

### 2.4. Bioinformatic Analysis of LrMYB30

Phylogenetic analysis of LrMYB30 was performed using MrBayes, with the resulting tree visualized and annotated via the Interactive Tree of Life (iTOL) web server (https://itol.embl.de, accessed on 28 November 2025). Protein secondary structure prediction was conducted through the Pfam database interface (http://pfam.xfam.org/search, accessed on 28 November 2025).

### 2.5. Subcellular Localization Assay for LrMYB30 Gene

To determine the subcellular localization of LrMYB30 from L. *ruthenicum*, a C-terminal fusion construct with eGFP was generated by cloning the *LrMYB30* gene into the pBI121-eGFP vector using specific restriction sites, such as XbaI and BamHI. For nuclear co-localization, a control plasmid expressing mCherry fused to a nuclear localization signal (NLS) was employed. Both constructs were initially propagated in *Escherichia coli* strain DH5α and subsequently transformed into *Agrobacterium tumefaciens* strain GV3101. *Nicotiana benthamiana* leaves were then agroinfiltrated with these bacterial cultures for transient expression. At 48–72 h post-infiltration, the GFP and mCherry fluorescence signals were visualized using fluorescence microscopy (Leica DM5000 B, Wetzlar, Germany). The localization pattern of LrMYB30-eGFP was directly compared with that of the NLS-mCherry nuclear marker.

### 2.6. Promoter Cloning

To identify regulatory regions, homology-based comparisons of candidate genes (*LrANR*, *LrLAR*, *LrUF3GT*, *LrDFR*, and *LrMYB30*) were conducted against the reference genomes of red-fruited *Lycium barbarum* and black-fruited *Lycium ruthenicum* using NCBI BLAST 2.17.0 (E-value < 1 × 10^−50^, query coverage ≥ 80%). Transcriptional start sites (TSS) were determined through integrative analysis of 5′-RACE mapping data and genomic annotations. For each gene, a ∼2000 bp upstream regulatory region was PCR-amplified from genomic DNA of L. *ruthenicum*, with primer pairs designed to target evolutionarily conserved motifs identified through multiple sequence alignment (Clustal Omega v1.2.4) of orthologous promoters.

Genomic DNA from black-fruit *Lycium ruthenicum*, extracted via the CTAB method, was used as the template for PCR amplification with high-fidelity DNA polymerase (PrimeSTAR^®^ Max, Takara, Tokyo, Japan). The PCR protocol consisted of: initial denaturation at 94 °C for 5 min; 35 cycles of denaturation at 94 °C for 30 s, annealing at [Tm] °C for 30 s, and extension at 72 °C for 2–3 min; followed by a final extension at 72 °C for 10 min. Amplified PCR products were resolved on 1% agarose gels, followed by gel extraction and purification prior to validation via Sanger sequencing. The correct promoter sequences were subsequently obtained. The type and location of cis-regulatory elements within the cloned promoter sequences were predicted using the PlantPAN 4.0 online database.

### 2.7. Yeast One-Hybrid Assay

Competent *E. coli* strain DH5α cells harboring pGADT7 or pHIS2 vectors were cultured in LB medium supplemented with appropriate antibiotics (ampicillin for pGADT7; kanamycin for pHIS2) for plasmid amplification at 37 °C. The CDS sequences of target genes (e.g., *LrMYB30*) and promoter fragments (e.g., *LrANRpro*, *LrLARpro*, *LrDFRpro* and *LrUF3GTpro*) were amplified via high-fidelity PCR using gene-specific primers with engineered restriction sites and genomic DNA as a template. The purified products were subjected to double restriction enzyme digestion, for instance with EcoRI and SacII, and ligated into linearized pGADT7 (prey vector) or pHIS2 (bait vector), respectively. The recombinant plasmids were constructed and transformed into *E. coli*, and correct recombinant plasmids were verified by colony PCR and extracted.

The constructed pGADT7-LrMYB30 was co-transformed with pHIS2-*LrANRpro* and pHIS2-*LrLARpro* into Y187 yeast competent cells. As a control, the empty vector pGADT7 was co-transformed with each pHIS2-promoter recombinant plasmid. The co-transformation mixtures were introduced into Y187 cells using the PEG/LiAc method, and positive transformants were selected on SD/–Trp/–Leu medium. To detect interactions and rule out self-activation, positive yeast cultures were grown to OD_600_ = 0.2, serially diluted (10^−1^ to 10^−3^), and spotted onto SD/–Trp/–Leu/–His triple-dropout medium containing 0, 30, 60, or 100 mM 3-AT. Cultures were incubated at 29 °C for 4 days, followed by visual assessment of colony growth.

### 2.8. Dual Luciferase (Dual-Luc) Assay

The full-length CDS of *LrMYB30* was subcloned into the binary overexpression vector pBI121 (35S promoter) using BamHI and SacII restriction sites. The promoter fragments of *LrDFR*, *LrANR*, *LrUF3GT*, *LrLAR*, and *LrMYB30* were cloned into the pGreenII0800-LUC reporter vector containing the Renilla luciferase (REN) internal control cassette. For *Agrobacterium-mediated* transient expression, the effector vector (pBI121-*LrMYB30*) and reporter vector (pGreenII0800-LUC) were separately introduced into Agrobacterium tumefaciens GV3101 and GV3101 (pSoup) using calcium chloride-mediated chemical transformation. Transformed colonies were selected on LB agar plates supplemented with 50 mg·L^−1^ rifampicin (Rif) and 50 mg·L^−1^ kanamycin (Kan), followed by culturing in liquid LB medium at 28 °C with orbital shaking at 180 rpm for 48 h.

Bacterial pellets were harvested by centrifugation (4000× *g*, 10 min), reconstituted in infiltration buffer (100 mM MgCl_2_, 10 mM MES, pH 5.6, 200 μM acetosyringone), and adjusted to OD_600_ = 0.5. After induction at 22 °C for 3–4 h in the dark, agroinfiltration was performed by mixing effector and reporter suspensions at a 4:1 ratio (*v*/*v*) and injecting into the abaxial epidermis of *Nicotiana benthamiana* leaves (3–5 th true leaves). Three biologically independent replicates were conducted with spatially separated leaf regions.

Leaf tissues were harvested 48 h post-infiltration, flash-frozen in liquid nitrogen, and stored at −80 °C until analysis. Protein extraction was carried out using 200 μL of 1× Passive Lysis Buffer (Promega, Madison, WI, USA) with 0.1 mm sterile zirconia beads (BioSpec Products, Bartlesville, OK, USA) through bead beating (FastPrep-24, MP Biomedicals, Irvine, CA, USA). Luciferase activities were quantified using the Dual-Luciferase^®^ Reporter Assay System (Promega, Madison, WI, USA) on a GloMax^®^ 96 Microplate Luminometer (Promega, Madison, WI, USA) following manufacturer instructions. Firefly luciferase (LUC) activity was normalized against Renilla luciferase (REN) internal control, with results expressed as relative luminescence units (RLU1/RLU2).

### 2.9. Virus-Induced LrMYB30 Gene Silencing

The CDS fragment of the *LrMYB30* gene that carrying the adaptor sequence was obtained by a pair of amplification primers (5′-ATGGCTGCATCTGTTGAAAAT; 3′-AATTTATTACTGCAGATTGTTGTTA), and the amplified product length is 1395 bp. The pTRV2 empty vector and the pTRV2-LIC vector ligated with *LrMYB30* were transformed into *Agrobacterium tumefaciens* strain GV3101 via heat shock. Transformed strains were inoculated into LB medium containing 50 mg/L Rif and 50 mg/L Kan and cultured overnight at 28 °C in 25 mL volumes. The following day, bacterial cells were harvested by centrifugation and resuspended in a solution containing 10 mM MES, 10 mM MgCl_2_, and 200 µM acetosyringone. After adjusting the OD600 to 1.0 and incubating at room temperature (25 °C) for 3–4 h, *Agrobacterium* carrying the pTRV1 vector was mixed with *Agrobacterium* carrying either the pTRV2 empty vector or the *LbNCED1*-pTRV2 vector at a 1:1 ratio for injection.

VIGS assays were conducted during the early fruit development phase, specifically at the green, immature fruit stage, to ensure temporal precision in gene function analysis (S2). The method of injection was as previously detailed by Li et al. [13] using a 1 mL syringe, the needle was positioned at the fruit growth point for pressurized delivery. For each treatment, injections were performed on at least three trees, each bearing at least 200 healthy fruits to ensure adequate sample representation. At 24 h after injection, fruits were collected, rapidly cryopreserved in liquid nitrogen, and maintained at −80 °C until subjected to biochemical or molecular analysis.

### 2.10. Real-Time PCR

Total RNA was isolated from fruit tissues using the RNAprep Pure Plant Kit (DP432; TianGen, Beijing, China), following the manufacturer’s protocol. The purity and quality of the RNA were confirmed by spectrophotometry, with A260/A280 and A260/A230 ratios of approximately 2.0 and 2.2, respectively. Genomic DNA was eliminated from RNA samples via treatment with RNase-Free DNase I (Takara, Dalian, China), according to the manufacturer’s protocol. Subsequently, cDNA was synthesized through reverse transcription reaction by a PrimeScript RT reagent kit (Takara, Dalian, China). To normalize gene expression data, *LrActin* was employed as an internal control, given its consistent transcriptional activity across diverse *Lycium* plant tissues, thereby providing a robust baseline for comparative analysis [33].

Quantitative real-time PCR was performed on a Bio-Rad CFX96 Touch™ Real-Time PCR System (Hercules, CA, USA), employing SYBR^®^ Premix Ex Taq™ (Takara Bio, Dalian, China) as the detection chemistry. Each 15 µL reaction contained 7.5 µL SYBR Premix Ex Taq mix, 1.0 µL cDNA template (containing 100 ng of cDNA), 1.2 µL primer mix (0.6 µL each of the forward and reverse primers) and 5.3 µL ddH_2_O. The amplification program was as follows: one cycle of 30 s at 95 °C, followed by 39 cycles of 5 s at 95 °C, 30 s at 60 °C and 30 s at 72 °C. The raw RT-qPCR data were acquired using Bio-Rad CFX Maestro™ Software V2.3 and were analyzed by the 2^−ΔΔCt^ method [34]. The primers used for RT-qPCR are described in Table A2, Appendix A.

### 2.11. Statistical Analysis

Statistical analyses were performed in IBM SPSS Statistics v25.0. One-way ANOVA was applied to evaluate significant differences among fruit developmental stages or treatment groups. The column figures were constructed by OriginPro 2024.

## 3. Results

### 3.1. *LrMYB30* Is a R3-Type Transcriptional Factor of MYB

**Figure 1:**
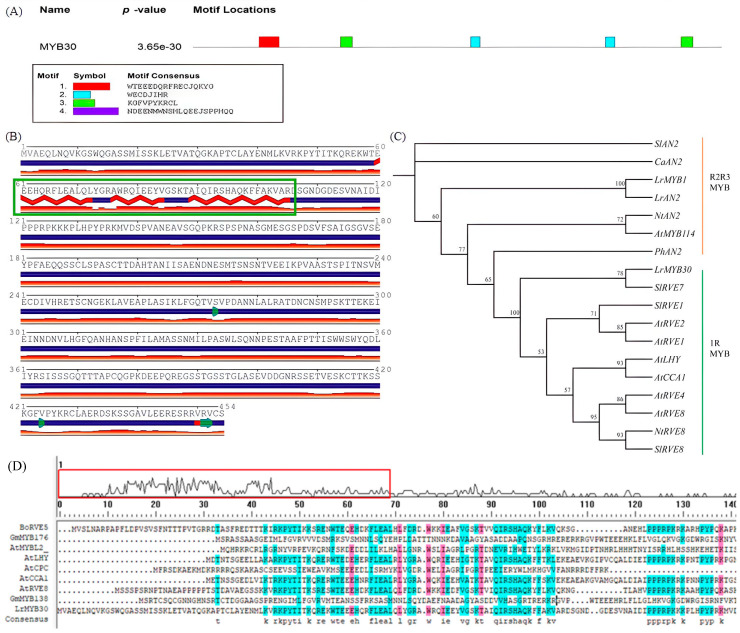
Bioinformatics analysis of the *LrMYB30* gene. (**A**) Structural map of the predicted motif gene distribution of LrMYB30; (**B**) sketch of the secondary structure of LrMYB30. The green boxes highlight three adjacent helix structures within each repeat, where the red diamond-shaped segments in the secondary structure diagram represent the helices. The green arrows indicate the C-terminal domain involved in transcriptional regulation; (**C**) phylogenetic analysis and evolutionary tree including LrMYB30; (**D**) Multiple sequence alignment of LrMYB30.

The full-length *LrMYB30* cDNA (GenBank: MN905500.1) was successfully cloned using Rapid Amplification of cDNA Ends (RACE) technology. SMART domain analysis (Figure 1) identified a single SANT/MYB DNA-binding domain spanning residues 50–104 in the N-terminal region, confirming its classification as an R3-MYB transcription factor. Notably, the N-terminal R2 MYB domain was partially truncated. While the conserved [D/E] Lx_2_[R/K]x_3_Lx_6_Lx_3_R motif within the R3 domain was preserved for bHLH protein interaction, no canonical ethylene-responsive element binding factor-associated amphiphilic repression (EAR) motif (“pdLNL[D/E] L” or “DLNxxP”) was detected downstream of the MYB domain. Additionally, the C-terminal region lacked the unique repression motif “TLLLFR” characteristic of CPC-type R3-MYBs. Phylogenetic analysis demonstrated that *LrMYB30* clustered separately from established R3-MYB-type anthocyanin biosynthesis repressors in other plant species, showing only distant homology (bootstrap support < 60%). This evolutionary divergence suggests functional specialization and species-specific regulatory mechanisms. These findings collectively indicate that *LrMYB30* does not belong to subgroup 4 R2R3-MYB repressors [17] or the CPC-type R3-MYB family, despite its longer amino acid sequence compared to typical R3-MYB proteins.

### 3.2. Subcellular Localization Analysis of *LrMYB30*

**Figure 2:**
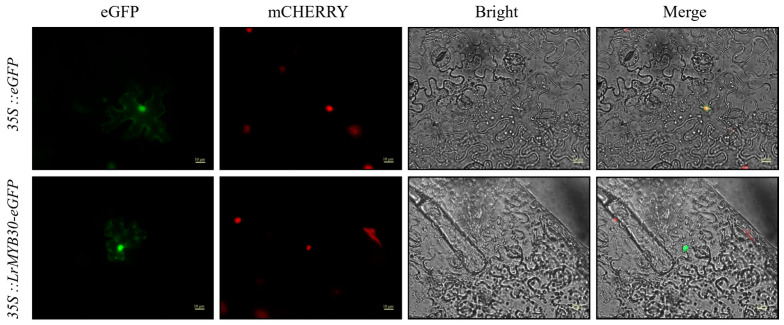
The subcellular localization of LrMYB30. The subcellular localization of LrMYB30 was assessed by transiently expressing an LrMYB30-eGFP fusion protein in *Nicotiana benthamiana* leaves via *Agrobacterium tumefaciens*-mediated transformation (strain GV3101). At 48–72 h post-infiltration, fluorescence microscopy was used to compare the fluorescence of LrMYB30-eGFP with that of a co-expressed NLS-mCherry nuclear marker.

To clarify the intracellular location where LrMYB30 exerts its function, a fusion expression vector pBI121-35S::*LrMYB30*-eGFP was constructed. Then, this fusion expression vector and the empty vector pBI121-35S::eGFP were, respectively, transformed into *Agrobacterium* GV3101. *Agrobacterium* carrying the nuclear localization signal NLS-mCherry was mixed with the *Agrobacterium* carrying the GFP signal vector at a ratio of 1:1 and then co-injected into the lower epidermis of *Nicotiana benthamiana* leaves. After dark culture for 2 to 3 days, the fluorescence signals were observed using a fluorescence microscope (Leica DM5000B). Figure 2 shows that through multi-channel observation, the eGFP fusion protein of *LrMYB30* mainly emits fluorescence in the nucleus and overlaps with the red nuclear localization fluorescence marker, indicating that LrMYB30 is mainly located in the nucleus and exerts its transcriptional regulatory function there.

### 3.3. Analysis of the Expression Characteristics of LrMYB30 in Fruits of Different Colors

**Figure 3:**
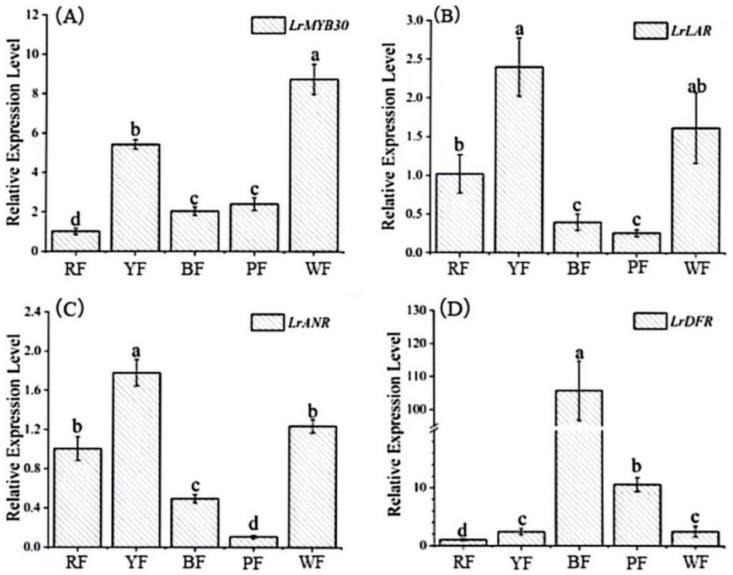
Relative expression levels of regulatory genes and structural genes of anthocyanin synthesis in fruits of different colors were quantified by RT-PCR. Fruit color abbreviations are: RF, red-fruit; YF, yellow-fruit; BF, black-fruit; PF, purple-fruit; WF, white-fruit. Relative expression of *LrMYB30* (**A**), *LrLAR* (**B**), *LrANR* (**C**) and *LrDFR* (**D**) in fruits of different colors. Columns in the graphs are means ± standard deviation of three biological replicates. Data with different letters between the same parameters indicate significant differences (*p* < 0.05), and the test of significance for post hoc multiple comparisons was performed using Duncan method.

To confirm the relationship between *LrMYB30* and fruits pigmentation, we used qRT-PCR technology to detect the relative expression levels of *LrMYB30* in *Lycium* fruits of different colors, as well as the relative expression levels of structural genes *LrDFR*, *LrANR*, and *LrLAR* in the anthocyanin metabolism pathway. Figure 3 shows that *LrDFR* has the highest relative expression level in black fruits, which have the highest anthocyanin accumulation, followed by purple fruits. On the contrast, the relative expression levels of *LrMYB30*, *LrANR*, and *LrLAR* are at a relatively low level in these fruits. It is known that white fruits are rich in proanthocyanidins but do not contain anthocyanins. Their expression levels of *LrMYB30*, *LrANR*, and *LrLAR* are high, while the relative expression level of *LrDFR* is lower than that in black and purple fruits. This is consistent with the transcriptome results conducted by Li et al. on the white fruits [32]. In yellow fruits, the relative expression levels of *LrMYB30*, *LrANR*, and *LrLAR* are also high, while red and yellow fruits do not accumulate anthocyanins or have very low accumulation levels. Among them, the relative expression level of *LrDFR* is at a low level. The results indicate that *LrMYB30* has similar expression characteristics in fruits that synthesize proanthocyanidins to the structural genes *LrANR* and *LrLAR*, while the relative expression pattern is opposite to that of *LrDFR*.

### 3.4. Effects of VIGS Mediated LrMYB30 Silencing on the Biosynthesis of Anthocyanins and PAs in Black Wolfberry

**Figure 4:**
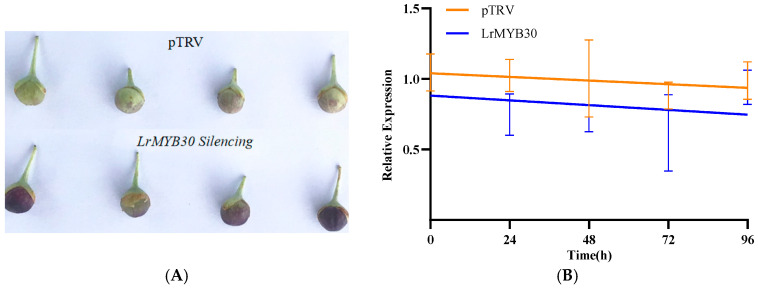

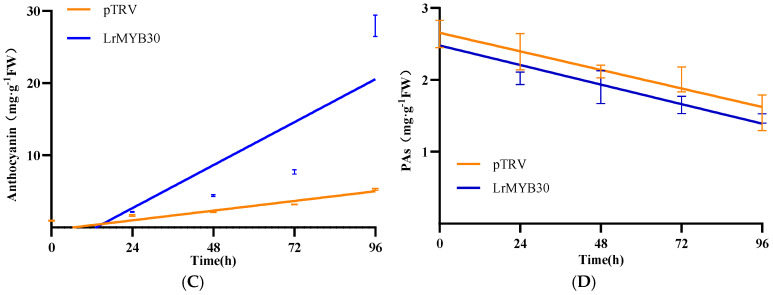
Effect of *LrMYB30* silencing on coloration of *Lycium barbarum* fruits. (**A**) Photographs of changes in fruit color in VIGS-*LrMYB30* group and pTRV2 group (control) after 48 h of silencing; (**B**) Relative expression of *LrMYB30* gene was measured in VIGS-*LrMYB30* group and pTRV2 group (control) within 96 h; (**C**) Anthocyanin level changes following *LrMYB30* silencing within 96 h; (**D**) PAs level changes following *LrMYB30* silencing within 96 h. Columns in the graphs display mean ± SD, computed from three independent biological replicates to ensure biological reproducibility.

The functions of *LrMYB30* were further validated in fresh black wolfberries using VIGS technology. It is shown that *LrMYB30* silencing increased anthocyanin content, but did not impact the structural genes expression involved in anthocyanin biosynthesis, e.g., *LrDFR* and *LrUF3GT*, as analyzed by RT-PCR. On the contrary, *LrMYB30* silencing down-regulated the expression of the structural genes that involved in PAs biosynthesis, e.g., *LrANR* and *LrLAR*, and thus reducing PAs content (Figure 4). These results indicate that *LrMYB30* exerts a negative effect on anthocyanin accumulation in fresh black wolfberries.

### 3.5. The Transcriptional Activation Effect of *LrMYB30* on PAs Biosynthesis Genes

**Figure 5:**
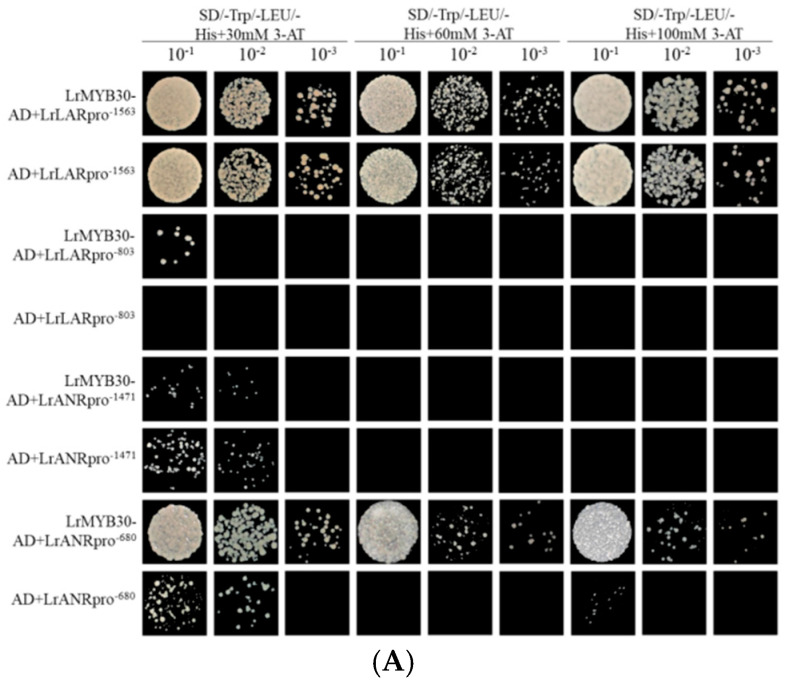

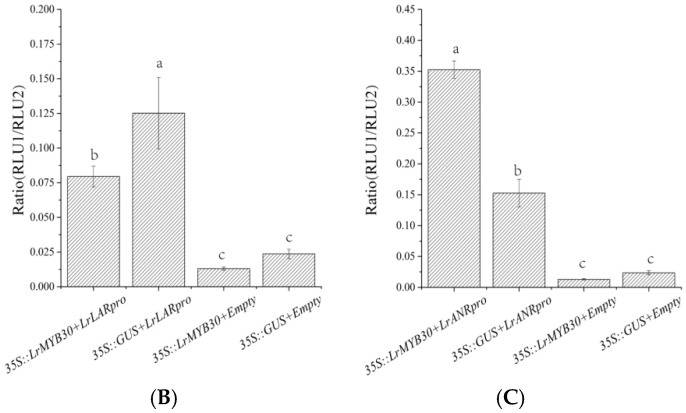
Interaction between LrMYB30 and the promoters of the pivotal genes *LrLAR* and *LrANR* for proanthocyanin synthesis. (**A**) Yeast one-hybrid detection of the interaction between LrMYB30 and the promoters of the core proanthocyanidin synthesis genes *LrLAR* and *LrANR*; (**B**) Dual-luciferase reporter assay to identify the activation/inhibition of *LrLAR* promoter by LrMYB30; (**C**) Dual-luciferase reporter Experimental detection of LrMYB30 activation/inhibition of *LrANR* promoter. The bars in the figure are the mean ± standard deviation of three biological replicates. Data with non-identical letters between the same parameters exhibit significant variation (*p* < 0.05), and the post hoc multiple comparison significance test method used Duncan’s method.

Based on the above observation, we hypothesized that LrMYB30 directly regulates the transcription of these PAs biosynthesis-related genes. To validate the proposed regulatory role, we performed a yeast one-hybrid (Y1H) assays to determine the physical association of LrMYB30 with the promoter elements of the relevant biosynthetic genes. As demonstrated in Figure 5, the Y1H assay validated that LrMYB30 could indeed bind to the promoters of *LrANR*, but did not bind to the promoters of *LrLAR*. To further substantiate the regulatory impact of LrMYB30 on its target genes, we performed transient co-expression assays in *N. benthamiana* leaves, employing a luciferase reporter system to quantify transcriptional activity. The results confirmed that LrMYB30 activated the promoter-driven reporter expression of *LrANR*. On the contrary, LrMYB30 fails to interact with the promoter sequences of key anthocyanin biosynthetic genes, e.g., *LrDFR* and *LrUF3GT*, nor modify them. These findings strongly support the interpretation that LrMYB30 acts as a negative regulator of anthocyanin biosynthesis by diverting the flavonoid metabolic pathway from anthocyanin synthesis to PAs synthesis at a later stage.

## 4. Discussion

The color transitions observed during berry ripening serve as natural indicators of ripening regulation and functional component dynamics [1]. Flavonoid compounds, particularly anthocyanins, represent both the primary pigments responsible for black wolfberry (*Lycium ruthenicum*) coloration and key functional metabolites [7,13]. The natural occurrence of diverse wolfberry colors (red, black, yellow, purple, and white) provides ideal materials for investigating anthocyanin metabolism, as well as the regulatory and evolutionary mechanisms underlying fruit coloration [25].

MYB-type transcription factors (TF) are among the most abundant TF families in plants, with their superfamily encompassing a large number of functionally diverse members involved in key regulatory processes [35]. MYB proteins typically contain two distinct regions: MYB TFs possess a conserved N-terminal DNA-binding domain (~52 amino acid residues) and a functionally flexible C-terminal region that mediates regulatory specificity through protein–protein interactions and target gene selection [17]. Based on the count of imperfectly repeated, evolutionarily retained motifs within the MYB domain, this family is categorized into four categories: 1R-, R2R3-, 3R-, and 4R-MYB proteins [19]. Among the aforementioned, R2R3-MYB TFs form the largest subfamily in plants, with 126 members identified in Arabidopsis thaliana and further grouped into 22 subgroups according to conserved motif patterns. Notably, several R2R3-MYB proteins act as activators of anthocyanin biosynthesis, as demonstrated by studies on *LrAN2* in black wolfberry [31,32,33,36].

In addition to these positive regulators, specific R2R3-MYB and R3-MYB proteins have been characterized as negative regulators of anthocyanin biosynthesis [37]. These repressors can inhibit MBW complex activity through two mechanisms: direct sequestration of essential TFs or indirect suppression of MBW component-encoding gene transcription [38,39,40,41,42,43,44]. Representative examples include the R3-MYB repressors such as PtMYBx from petunia and CPC from Arabidopsis, which competitively bind to bHLH partners and/or anthocyanin biosynthetic gene promoters against R2R3-MYB activators [38,39].

In this study, we identified *LrMYB30*, a R3-MYB TF from black wolfberry, that exhibits a novel regulatory function by redirecting the flavonoid metabolic flux from anthocyanin biosynthesis toward proanthocyanidin (PA) synthesis during late ripening stages. This metabolic shift effectively reduces anthocyanin accumulation and delays fruit coloration/ripening progression.

Previous studies have demonstrated that competition between anthocyanin and flavonol biosynthetic pathways generates floral pigment pattern variations across Mimulus species [45]. More recently, Lin et al. [46] reported that exogenously applied PAs can delay fruit coloring and softening in non-climacteric fruits. These findings collectively suggest that metabolic competition between anthocyanins and PAs for shared precursors constitutes a critical regulatory mechanism governing color development and ripening in non-climacteric fruits.

Compared to the well-established engineering of anthocyanin production, attempts to manipulate PA biosynthesis have yielded limited success, primarily resulting in increased anthocyanin levels or minor PA accumulation [47]. This bottleneck likely stems from insufficient understanding of PA biosynthetic complexity and its regulatory networks. Our identification of *LrMYB30* as a key modulator that spatially and temporally controls the anthocyanin/PA metabolic balance provides new insights into this regulatory puzzle, and this gene is expected to become an important resource for future PA metabolic engineering endeavors.

## Figures and Tables

**Figure A1:**
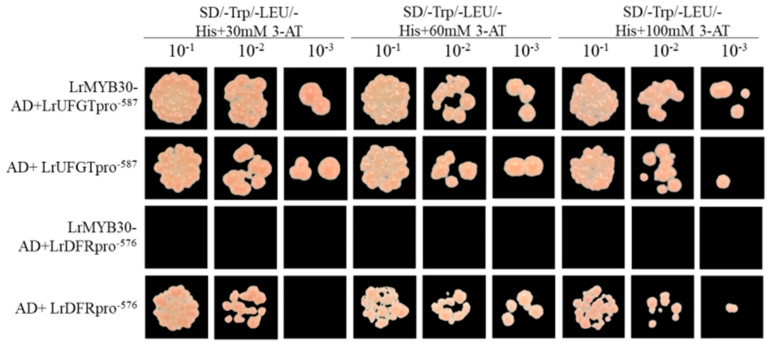
Yeast one-hybrid detection of the interaction between LrMYB30 and the promoters of the key anthocyanin synthesis genes *LrUFGT* and *LrDFR*. The extended plate incubation, necessary because of the weak initial signal, caused the yeast cells to develop a light red coloration, which is a characteristic phenotypic response to long-term culture.

## Data Availability

The original contributions presented in the study are included in the article. Further inquiries can be directed to the corresponding author.

## Appendix A

**Table A1:** The amplification primers for *LrMYB30* coding sequence (used for subcellular localization analysis).

Primer Name	Sequence (5′-3′)	Length (bp)
gfp-MYB30F	AGAACACGGGGGACTCTAATGGTAGCAGAACAACTGAA	35
gfp-MYB30R	GGTGGCGACCGGTACCCGTGAACACACACGGACCCTTC	37

**Table A2:** Real-time quantitative PCR primers.

Gene Name	Primer Direction	Sequence (5′-3′)	Length (bp)	Amplicon Length (bp)
LrActin	F	CTCAGCACCTTCCAGCAGAT	20	162
LrActin	R	TAACACTGCAACCGCATTTC	20	162
LrMYB30	F	TGCCAAGGTTGCTCGAGATT	20	137
LrMYB30	R	CCTCGTTAGCTACAGGGGAATC	22	137
LrDFR	F	TCTGTTAGAATTGCCGAAAG	20	133
LrDFR	R	CTCGAAATCCATAGGTGTTG	20	133
LrLAR	F	AAGTGTGGTTGCAGCTTTGACA	22	174
LrLAR	R	ATTTTGGGCATGTCATCCATCT	22	174
LrANR	F	CCTCTCTCATGGCTGGTCCTTA	22	165
LrANR	R	GGGCACGACAAACATCCTCTAC	22	165

**Table A3:** *LrMYB30* silencing sequence amplification primers.

Primer Name	Sequence (5′-3′)	Length (bp)
LrMYB30-F	CGACGACAAGACCCTATGGTAGCAGAACAACTGAA	35
LrMYB30-R	GAGGAGAAGAGCCCTCTATGAACACACACGGACCC	35

**Table A4:** The amplification primers for promoter sequence.

Primer Name	Sequence (5′-3′)	Length (bp)
UF3GTproR	CTCAGCACCTTCCAGCAGAT	20
UF3GTproF	TAACACTGCAACCGCATTTC	20
DFRproR	TGCCAAGGTTGCTCGAGATT	20
DFRproR	CCTCGTTAGCTACAGGGGAATC	22
ANRproF	TCTGTTAGAATTGCCGAAAG	20
ANRproR	CTCGAAATCCATAGGTGTTG	20
LARproR	AAGTGTGGTTGCAGCTTTGACA	22
LARproF	ATTTTGGGCATGTCATCCATCT	22

**Table A5:** Primers for yeast one hybrid experiment.

Primer Name	Primer Sequence (5′-3′)	Length (bp)
UFHIS	GACTCACTATAGGGCTTTCTAGACAAGTTCACCTACAT	38
URHIS	TCGCGAACGCGTGAGCTCTGTTCAAATGTTGGTTTAGGCT	40
DFRFHIS	GACTCACTATAGGGCGATCTCCAAAATTGTGTTGCAC	37
DFRRHIS	TCGCGAACGCGTGAGCTCTGTCTTTATACTGCTTGCACGT	40
ANRFHIS	GACTCACTATAGGGCTGGTTAATCATGGTGGACTCA	36
ANRRHIS	TCGCGAACGCGTGAGCTCCAGAAGTGGGGAAGACAACT	38
LARFHIS	GACTCACTATAGGGCGCTGATGGCGTGTGATCTAT	35
LARRHIS	TCGCGAACGCGTGAGCTCATTGGTTTGGAGGTGATGGT	38
pHIS-M3PRO-F	TCACTATAGGGCGAATTCGGGTTAAGGGTCTTGGTGTC	38
pHIS-M3PRO-R	TCGCGAACGCGTGAGCTCATAGTTGGAATCGAAACCTC	38
pHIS-A2PRO-F	TCACTATAGGGCGAATTCCGTGTGCATAAGATGGGAAA	38
pHIS-A2PRO-R	TCGCGAACGCGTGAGCTCGACCGCGATTCATTCTTATG	38
MYB-AD-F	GGAATTCCATATGATGGTAGCAGAACAACTGAA	33
MYB-AD-R	CCGGAATTCTCAACTATGAACACACACGG	29

**Table A6:** Dual-LUC experimental primers.

Primer Name	Primer Sequence (5′-3′)	Length (bp)
UFG	CACTATAGGGCGAATTGTTTCTAGACAAGTTCACCTACAT	40
URG	GCCGCTCTAGAACTAGTTGTTCAAATGTTGGTTTAGGCT	39
DFG	CACTATAGGGCGAATTGGATCTCCAAAATTGTGTTGCAC	39
DRG	GCCGCTCTAGAACTAGTTGTCTTTATACTGCTTGCACGT	39
RFG	CACTATAGGGCGAATTGTGGTTAATCATGGTGGACTCA	38
RRG	CCGCTCTAGAACTAGTCAGAAGTGGGGAAGACAACT	36
LFG	CACTATAGGGCGAATTGGCTGATGGCGTGTGATCTAT	37
LRG	GCCGCTCTAGAACTAGTATTGGTTTGGAGGTGATGGT	37
PG-A2PRO-F	ACTATAGGGCGAATTGCGTGTGCATAAGATGGGAAA	36
PG-A2PRO-R	GCCGCTCTAGAACTAGTGACCGCGATTCATTCTTATG	36
PG-M3PRO-F	CACTATAGGGCGAATTGGGGTTAAGGGTCTTGGTGTC	36
PG-M3PRO-R	GCCGCTCTAGAACTAGTATAGTTGGAATCGAAACCTC	36
pBI-MYB30F	CGGGTACCGGTCGCCACCATGGTAGCAGAACAACTGAA	38
pBI-MYB30R	GATCGGGGAAATTCGTCTATGAACACACACGGACCC	36
